# Inhibitory effects of *Euphorbia supina* on *Propionibacterium acnes*-induced skin inflammation in vitro and in vivo

**DOI:** 10.1186/s12906-018-2320-8

**Published:** 2018-09-27

**Authors:** Hyeon-Ji Lim, Yong-Deok Jeon, Sa-Haeng Kang, Min-Kyoung Shin, Ki-Min Lee, Se-Eun Jung, Ji-Yun Cha, Hoon-Yoen Lee, Bo-Ram Kim, Sung-Woo Hwang, Jong-Hyun Lee, Takashi Sugita, Otomi Cho, Hyun Myung, Jong-Sik Jin, Young-Mi Lee

**Affiliations:** 1 Department of Oriental Medicine Resources, Chonbuk National University, 79 Gobongro, Iksan, Jeollabuk-do 54596 South Korea; 2 Department of Oriental Pharmacy, College of Pharmacy, Wonkwang-Oriental Medicine Research Institute, Wonkwang University, Iksan, Jeollabuk-do 54538 South Korea; 3 Department of Pharmacy, College of Pharmacy, Dongduk Woman’s University, 23-1 Wolgok-Dong, SungBuk-Gu, Seoul, 02748 South Korea; 4 Department of Microbiology, Meiji Pharmaceutical University, 2-522-1 Noshio, Kiyose, Tokyo, 204-8588 Japan; 5 Department of Ecology Landscape Architecture-Design, College of Environmental and Bioresource Sciences, Chonbuk National University, Iksan, South Korea

## Abstract

**Background:**

*Euphorbia supina* (ES) plant has been used as treatment for inflammatory conditions. The antibacterial effect and the anti-inflammatory mechanism of ES for *Propionibacterium* (*P.*) *acnes*-induced inflammation in THP-1 cells and acne animal model remain unclear. Therefore, the objective of the present study was to determine the antibacterial and anti-inflammatory activities of ES against *P. acnes*, the etiologic agent of skin inflammation.

**Method:**

The antibacterial activities of ES were tested with disc diffusion and broth dilution methods. Cytotoxicity of ES at different doses was evaluated by the MTT assay. THP-1 cells were stimulated by heat-killed *P. acnes* in the presence of ES. The pro-inflammatory cytokines and mRNA levels were measured by ELISA and real-time-PCR. MAPK expression was analyzed by Western blot. The living *P. acnes* was intradermally injected into the ear of BLBC/c mice. Subsequently, chemical composition of ES was analyzed by liquids chromatography-mass spectrometry (LC-MS).

**Result:**

ES had stronger antibacterial activity against *P. acnes* and inhibitory activity on lipase. ES had no significant cytotoxicity on THP-1 cells. ES suppressed the mRNA levels and production of IL-8, TNF-a, IL-1β in vitro. ES inhibited the expression levels of pro-inflammatory cytokines and the MAPK signaling pathway. Ear thickness and inflammatory cells were markedly reduced by ES treatment. Protocatechuic acid, gallic acid, quercetin, and kaempferol were detected by LC-MS analysis in ES.

**Conclusions:**

Our results demonstrate antibacterial and anti-inflammatory activities of ES extract against *P. acnes*. It is suggested that ES extract might be used to treatment anti-inflammatory skin disease.

**Electronic supplementary material:**

The online version of this article (10.1186/s12906-018-2320-8) contains supplementary material, which is available to authorized users.

## Background

Acne, one of the most common skin diseases, affects more than 80% of all adolescents [1]. Acne is an inflammatory disorder of the pilosebaceous unit characterized by excessive sebum production, follicular hyperkeratinization, and colonization of *Propionibacterium* (*P.*) *acnes* [2], a Gram-positive anaerobic bacterium. It has been reported that *P. acnes* is a major factor in acne inflammatory reaction by activating toll-like receptors TLR2 and TLR4 [3]. During acne inflammatory reaction, *P. acnes* induces the production of pro-inflammatory cytokines such as interleukin (IL)-1β, IL-6, IL-8, and tumor necrosis factor (TNF)-α in monocytes and keratinocytes [4, 5]. IL-8, a CXC chemokine, is a strong proinflammatory chemotactic factor for lymphocytes, basophils, and neutrophils. It is increased in keratinocytes by *P. acnes* stimulation [6]. Therefore, suppression of *P. acnes*-induced inflammatory cytokine is one of the major targets for treating acne inflammation.

*Euphorbia supina* (ES) plant has been used for traditional formulations of herbal medications. It has various pharmacological effects, including anti-oxidant, anti-arthritic, detoxification, diuretic, and hemostatic effects in various cell types [7]. ES contains a number of biologically organic substances including tannins, terpenoids, and polyphenols [8]. Recent studies have shown that ES possesses antibacterial activity against *Staphylococcus aureus* [9]. It can also inhibit cancer cell proliferation of U937 human leukemic cells [10]. However, the antibacterial effect and anti-inflammatory mechanism of ES in *P. acnes*-induced inflammation in vitro and in vivo remain unclear. Therefore, the objective of this study was to determine the antibacterial and anti-inflammatory effects of ES in an in vitro model using heat-killed *P. acnes* and living *P. acnes*-induced acne skin disease model.

## Methods

### ES preparation

The dried ES was purchased from Wonkwang PHARMACEUTICAL CORPORATION (Iksan, Korea). ES was washed twice with distilled water followed by drying, then extracted with 70% ethanol at room temperature for 3 days. The extract was concentrated with a vacuum evaporator and stored at 4 °C before experiments. The yield of ES extract was 6.14%. A voucher specimen (JUHES-1660) has been deposited at Department of Oriental Medicine Resources, Chonbuk National University (Iksan, Korea).

### Preparation of *P.acnes*

*P. acnes* (KCTC 3315, Daejeon, Korea) was obtained from the Korean Collection for Type Culture (KCTC, Daejeon, Korea) and grown under anaerobic condition in 10 ml of GAM (Nissui Pharmaceutical, Japan) liquid medium at 37 °C for OD600 = 1.0 (logarithmic growth phase). A total cell count of 10 ml of *P.acnes* suspension was approximately 1.34 × 10^9^ colony forming unit (CFU). *P. acnes* were harvested by centrifugation at 4000 rpm for 15 min at 4 °C to remove supernatant. Bacterial pellets were washed three times with 10 ml of PBS and finally suspended in 1 ml of PBS. The *P. acnes* suspension was incubated at 80 °C for 30 min for heat-killing reaction. To use cell stimulation heat-killed *P. acnes* suspension was stored at 4 °C until use. To use in vivo experiment living *P. acnes* suspension was stored at − 80 °C until use.

### Antibacterial assay

ES was dissolved in dimethyl sulfoxide (DMSO) at different concentrations (100, 200 mg/ml). Each concentration of ES was then impregnated onto a paper disc (8 mm in diameter) and placed on the top of GAM agar plate containing 100 μl of bacterial solution containing *P. acnes*. These plates were incubated at 37 °C for 48 h under anaerobic condition. Tetracycline was employed as a positive control. Minimum inhibitory concentration (MIC) test was performed in sterile 96-well plates using broth dilution method. Briefly, bacteria were cultured to stationary phase for 48 h at 37 °C. The turbidity and cell numbers were measured 0.418 at 620 nm and 1.64 × 10^7^ CFU, respectively. The cultivated bacteria was added into microplate at 0.5% of total volume (200 μl). ES extract was adjusted to concentrations through serial dilution in culture medium into 0 to 9 mg/ml. After incubating at 37 °C in an anaerobic jar for 48 h, the turbidity was obtained on a microplate ELISA reader as an indicator of bacterial growth.

To test minimum bactericidal concentration (MBC), 1 μl of various concentrations of the ES extract mixed with diluted solution of *P. acnes* for 48 h, 37 °C. And then MBC was performed by sub culturing the MIC dilutions on the sterile GAM agar broth. The lowest concentration of the extract in which bacteria failed to grow (99% no growth) was reported as MBC.

### Lipase activity

*P.acnes* was grown in brain heart infusion (BHI) broth, and 100 μl amounts of cell suspensions (5.0 × 10^8^ cells/mL) in BHI broth with final concentrations of 0.01, 0.1, 1, 10, 100 μg/mL ES were added to wells of 96well plates. The plates were anaerobically incubated at 37 °C for 24 h. Fifty microliter amounts of supernatants were centrifuged and the supernatants mixed with 50 μl of 10 mM 4-methyl umbelliferyl oleate (4-MUO) (Sigma Aldrich, St. Louis, USA) dissolved in 13 mM Tris-HCL, 0.15 M NaCl, and 1.3 mM CaCl_2_ (pH 8.0). The mixtures were incubated for 30 min at 25 °C under light illumination. Enzymatic reactions were terminated by adding 100 μl of 0.1 M sodium citrate (pH 4.2). The levels of 4-methylumbelliferone released by the lipase were measured using a fluorometric microplate reader (Fluoroskan Ascent™; Thermo Fisher Scientific, MA, USA); the excitation wavelength was 355 nm and the emission wavelength was 460 nm.

### Cell viability assay

Human monocyte THP-1 cells were maintained in RPMI 1640 (Gibco, Carlsbad, CA, USA) supplemented with 10% fetal bovine serum (FBS, WELGENE, South Korea) and 1% penicillin (Gibco, USA) at 37 °C in an atmosphere with 5% CO_2_.

MTT assay was performed to measure cell viability. Briefly, THP-1 cells (3.0 × 10^4^ cells/well) was incubated with various concentration of ES (0.1–10 μg/ml) for 24 h. MTT solution (500 μg/ml) was then added to each well and incubated at 37 °C for 8 h. Formazan crystal produced by living cell was dissolved in DMSO. The absorbance of each well was measured at wavelength of 540 nm on a microplate ELISA reader.

### Enzyme-linked immunosorbent assay (ELISA)

THP-1 cells (3.0 × 10^5^ cells/well) were pre-treated with indicated concentrations of ES (0.1–10 μg/ml) for 1 h followed by stimulation with heat-killed *P. acnes* for 18 h. The levels of IL-1β, IL-8, and TNF-α in culture media were measured with an ELISA kit (BD Pharmingen, San Diego, CA, USA). The absorbance of the ELISA plate was measured at wavelength of 405 nm using an automated microplate ELISA reader.

### RNA isolation and real- time RT PCR

Total cellular RNA was isolated from human monocyte THP-1 cells using easy-BLUE reagent Kit (iNtRON Biotechnology, Seoul, South Korea). Total RNA was used as template for first-strand cDNA synthesis using a Power cDNA Synthesis Kit (iNtRON Biotechnology, Seoul, South Korea) according to the manufacturer’s instructions. The transcription levels of genes were determined with a StepOnePlus Real-time PCR System (Applied Biosystems, Foster City, CA, USA). The relative gene expression was calculated using the comparative CT method with StepOne Software v2.1 (Applied Biosystems, Foster City, CA, USA). The expression of β-actin mRNA was used as an endogenous control. We used TNF-Forward primer 5’-TTACGCCTTTGAAGTTAGCAG-3′ and TNF-Reverse primer 5’-CGTCCAAATACATCGCAAC-3′ for TNF-α, 5′- TCTTTGAAGAAGAGCCCGTCCTC- 3′ /5’-GGATCCACACTCTCCAGCTGCA- 3′ for IL-1β, and 5′- GAATACTCTATTGCCGATGGT-3′/5’-CGATGGGTTTGCGTTTG-3′ primers for β-Actin as an internal control.

### Western blot analysis

Stimulated cells were rinsed with ice-cold PBS and lysed using lysis buffer (iNtRon Biotech, Seoul, South Korea) for 1 h. Total cell lysates were centrifuged at 12,000×g at 4 °C for 10 min to obtain supernatants. After bicinchoninic acid (BCA, Sigma) protein quantification assay, the supernatant was mixed with 2× sample buffer, boiled at 95 °C for 5 min, separated by 10% SDS-polyacrylamide gel electrophoresis, and transferred to nitrocellulose membrane (Roche Diagnostics, IL, US). These membranes were blocked with 5% skim milk in PBS-Tween-20 (PBST) for 1 h at room temperature followed by overnight incubation with anti-phospho-JNK, anti-p38, and anti-ERK antibodies at 4 °C. After washing three times with PBST, these membranes were incubated with secondary antibodies for 1 h at room temperature followed by three times of washes with PBST. The protein-antibody complexes were visualized with ECL Western blotting Luminol Reagent (Santa Cruz Biotech, CA, USA). Images were recorded with an LAS-4000 image reader (Fujifilm Life Sciences, Tokyo, Japan).

### Experimental animal model

All experimental protocols (CBNU2016–085) were approved by the Committee on the Care of Laboratory Animal Resources, Chonbuk National University and were conducted in accordance with the Guide for the Care and Use of Laboratory Animals. Male BALB/c mice (6 weeks old) were obtained from SAMTAKO (Osan, South Korea). They were individually housed in polycarbonate cages and maintained under constant temperature (25–27 °C) with a 12 h light-dark cycle. They were provided free access to standard diet and tap water. These animals were allowed to acclimate to these conditions for at least 7 days before the experiment.

These mice were randomly divided into 4 different groups (4 mice/group) as follows: B: non-treatment, PA: Live *P. acnes* (1.34 × 10^9^ CFU/ 20 μl PBS) was intradermally injected into the left ear. The right ear was received an equal amount of PBS. PA/ES 1 mg and PA/ES 10 mg with live *P. acnes* were intradermal injected into both the left and right ears. At 24 h after the injection, ES (1 or 10 mg/ml in PBS) was applied to the surface of the right ear skin of each group. At the end of each treatment period, these animals were sacrificed by cervical dislocation and their ears were measured using a micro-caliper (Mitutoyo, Kanagawa, Japan).

### Histological analysis

Ear section sample was fixed with 10% formaldehyde, embedded in paraffin wax, routinely processed and sectioned into 4-μm-thick slices. These ear sections were stained with hematoxylin and eosin (H&E) followed by examination with a light microscope to determine the presence of edema and inflammatory cell accumulation.

### HPLC-MS

The extract of ES was dissolved in MeOH into 0.1 mg/ml. Gallic acid (Sigma aldrich chemie GmbH, Germany), protocatechuic acid (Hwi analytik GmbH, Germany), quercetin (Tokyo Chemical Industry, Tokyo, Japan) and kaempferol (Santa Cruz Biotechnology Inc., USA) were dissolved in MeOH for analysis, either. HPLC was performed on an Agilent 1100 system (Agilent Technologies, Waldbronn, Germany) with a photodiode array detector DAD (G1315D) and Agilent 1100 series quard pump (G1311A), and an Agilent 6410 Triple Quadrupole LC/MS mass spectrometer (Agilent Technologies, Waldbronn, Germany) coupled with an ESI (electrospray ionization) interface and an ion trap mass analyzer. The ESI (electrospray ionization) source was operated in negative ionization modes. Analysis of included compounds were performed under the following conditions: column, TSK-gel ODS-80Ts (Tosoh Co., Tokyo, Japan 4.6 mm X 150 mm); mobile phase, 0.1% formic acid (solvent system A) and CH_3_CN (solvent system B) in a gradient mode (B from 20 to 80% in 30 min); sample injection, 5 μl; flow rate, 0.5 ml/min; temperature, 30 °C, UV wavelength, 254 nm and 350 nm. High-purity nitrogen was used as dry gas at a flow rate at 10 L/min, gas temperature at 300 °C; fragmentor voltage 150 V. Nitrogen was used as nebulizer at 30 psi and capillary voltage, ±4000 V.

### Statistical analysis

All results are presented as mean ± S.E.M. Results were analyzed using Graph Pad Prism version 5.0 program (Graph Pad Software, Inc., La Jolla, CA, USA). One-way analysis of variance with Tukey hoc post test was used to determine the differences. Statistical significance was considered when *P* value was less than 0.05.

## Result

### Anti-bacterial activity of ES against *P. acnes*

**Fig. 1:**
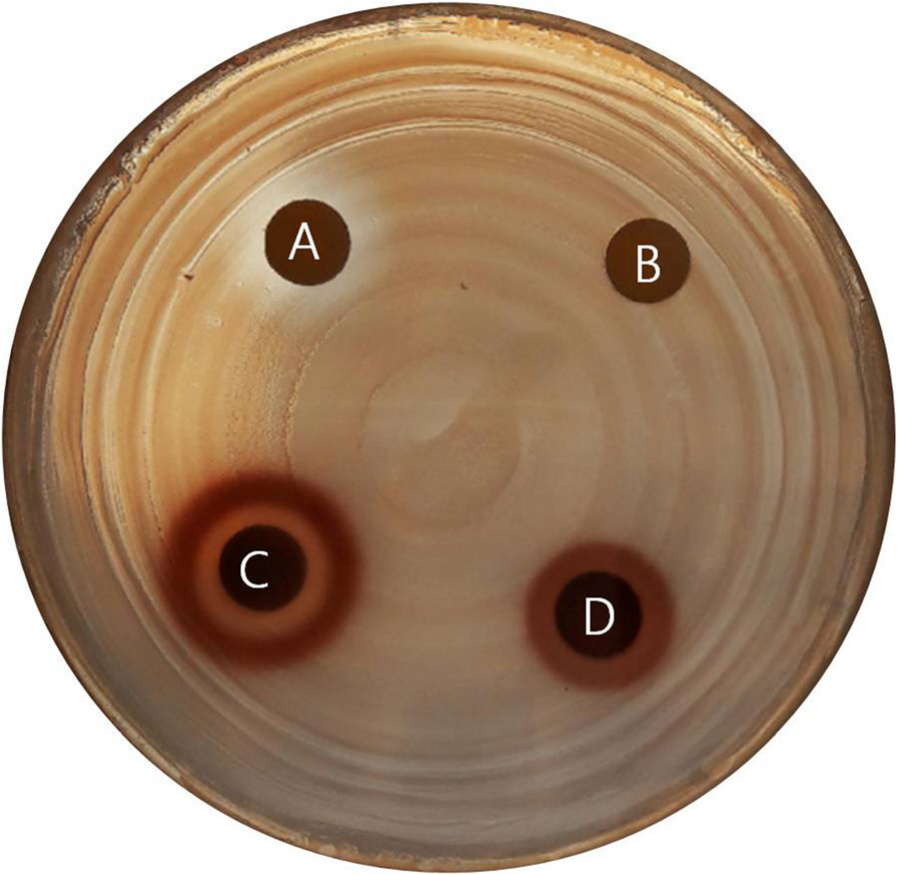
Antibacterial activity of ES against *P. acnes* (**a**; tetracycline 50 μg/ml, **b**; DMSO, **c**; ES 200 mg/ml, **d**; ES 100 mg/ml)

To evaluate the antibacterial activity of ES extract against *P. acnes* growth, bacteria was co-cultured with various concentrations of ES for 48 h. The MIC value of ES was determined to be 3.0 mg/ml. The MBC value of ES was found to be 7.0 mg/ml. We further performed disc diffusion assay using DMSO as a negative control and tetracycline as a positive control. ES ethanol extracts at concentrations of 100 mg/ml, 200 mg/ml, resulted in clear zones of 9.0 mm, 14.0 mm diameter, respectively (Fig. 1). However the lower concentration of ES (50 mg/ml, 20 mg/ml, and 10 mg/ml) had no antibacterial activity against *P. acnes* (data was not shown). In addition, ES had antibacterial activity against other skin microbes such as *Propionibacterium granulosum* (*P. granulosum*), *Staphylococcus aureus* (*S. aureus*), and *Staphylococcus epidermis* (*S. epidermis*) in concentration of 200 mg/ml (Additional file 1: Figure S1). In addition, ES has effect of lipase inhibition on *P.acnes*. The production of lipase on *P.acnes* was reduced by ES treatment (59.88 ± 6.52% on ES 100 μg/ml treatment) (Table 1.).

**Table 1:** The inhibitory effect of ES on lipase activity. P.acnes (5.0 × 108 cells/ ml) and 0.01 –.100 μg/ml of ES were added to 96 well plates. 24 h later, 50 μl of supernatants were mixed with 50 μl of 4-MUO. 30 min later, 100 μl of 0.1 M sodium citrate was added. Then lipase.activity was measured using fluorometric microplate reader. Values represent mean ± SD (*n* =.4). Data were analyzed by Tukey post hoc test (*P < 0.05 versus *P.acnes* alone)

		Inhibition (%)	SD
*P.acnes* (5 × 108 CFU)	ES 100 μg/ml	59.88	6.52*
*P.acnes* (5 × 108 CFU)	ES 10 μg/ml	2.20	5.38
*P.acnes* (5 × 108 CFU)	ES 1 μg/ml	5.00	8.38
*P.acnes* (5 × 108 CFU)	ES 0.1 μg/ml	0.53	2.34
*P.acnes* (5 × 108 CFU)	ES 0.01 μg/ml	0.75	3.34
*P.acnes* (5 × 108 CFU)	ES 0 μg/ml		

### Effects of ES on heat-killed *P. acnes-*induced pro-inflammatory cytokines in THP-1 cells

**Fig. 2:**
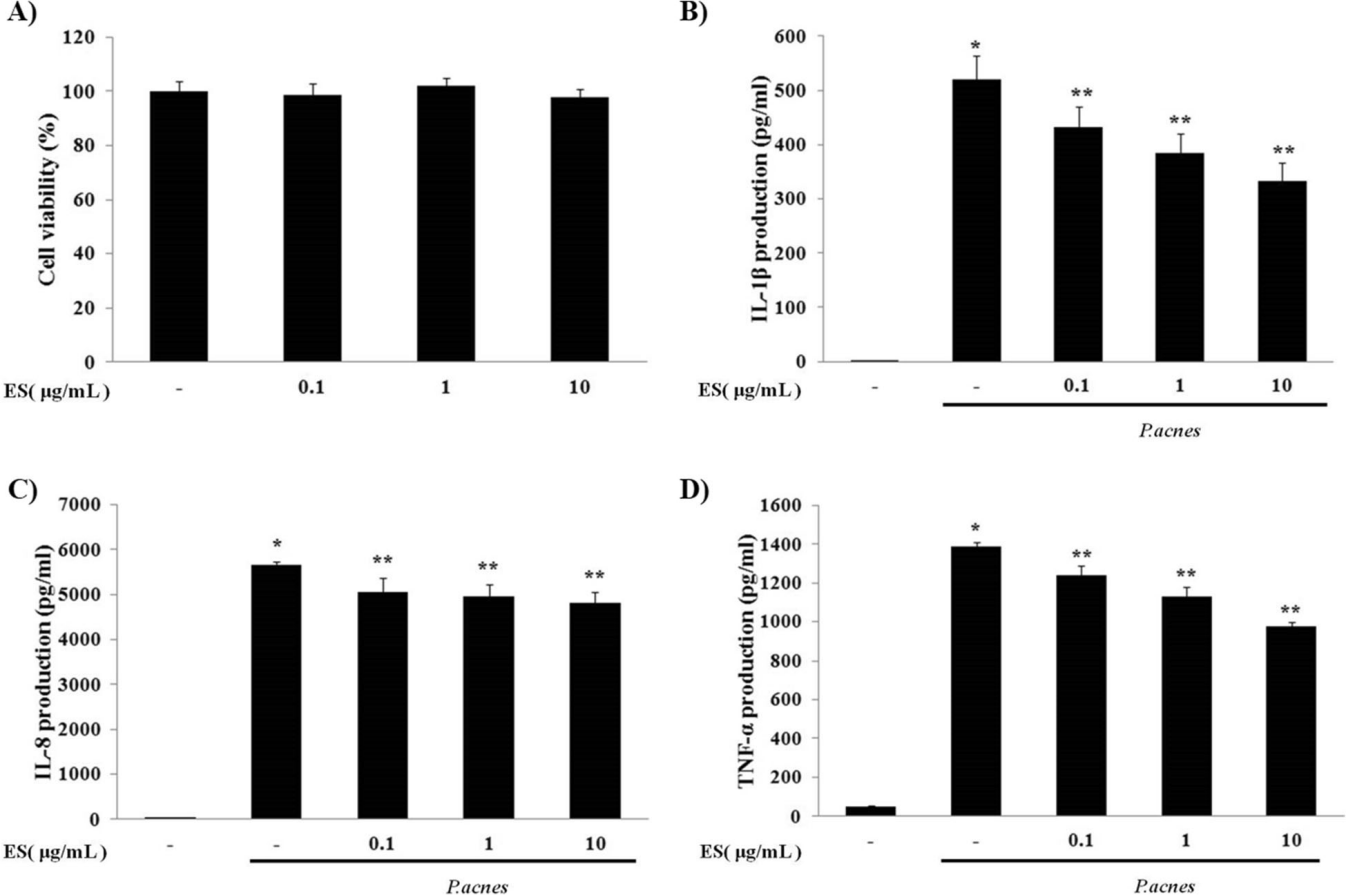
Effect of ES on cell viability and *P. acnes*-induced pro-inflammatory cytokines in THP-1 cells. (**a**) Cell viability of ES was determined by MTT assay in THP-1 cells. THP-1 cells were treated with 0.1, 1 and 10 μg/ml of ES for 24 hrs. ELISA results demonstrate that ES suppressed the secretion of (**b**) IL-1β, (**c**) IL-8 and (**d**) TNF-α in *P. acnes*-stimulated THP-1 cells. Values represent mean ± S.E.M. Data were analyzed by Tukey post hoc test ( * *p* < 0.05 versus control and ** *p* < 0.05 versus *P.acnes* alone)

Cell viability of THP-1 cells was determined by MTT assay. THP-1 cells were treated with various concentrations of ES (0.1, 1, or 10 μg/ml) for 24 h. ES had no significant cytotoxicity on THP-1 cells (Fig. 2a). After treatment with ES, the suppressive effect of ES on heat-killed *P. acnes-*stimulated inflammatory cytokine secretion was determined. ES suppressed the secretion of TNF-α, IL-1β, and IL-8 in THP-1 cells treated with heat-killed *P. acnes*. These results suggest that ES could effectively inhibit pro-inflammatory cytokine secretion in *P. acnes*-stimulated THP-1 cells (Fig. 2b, c, d).

**Fig. 3:**
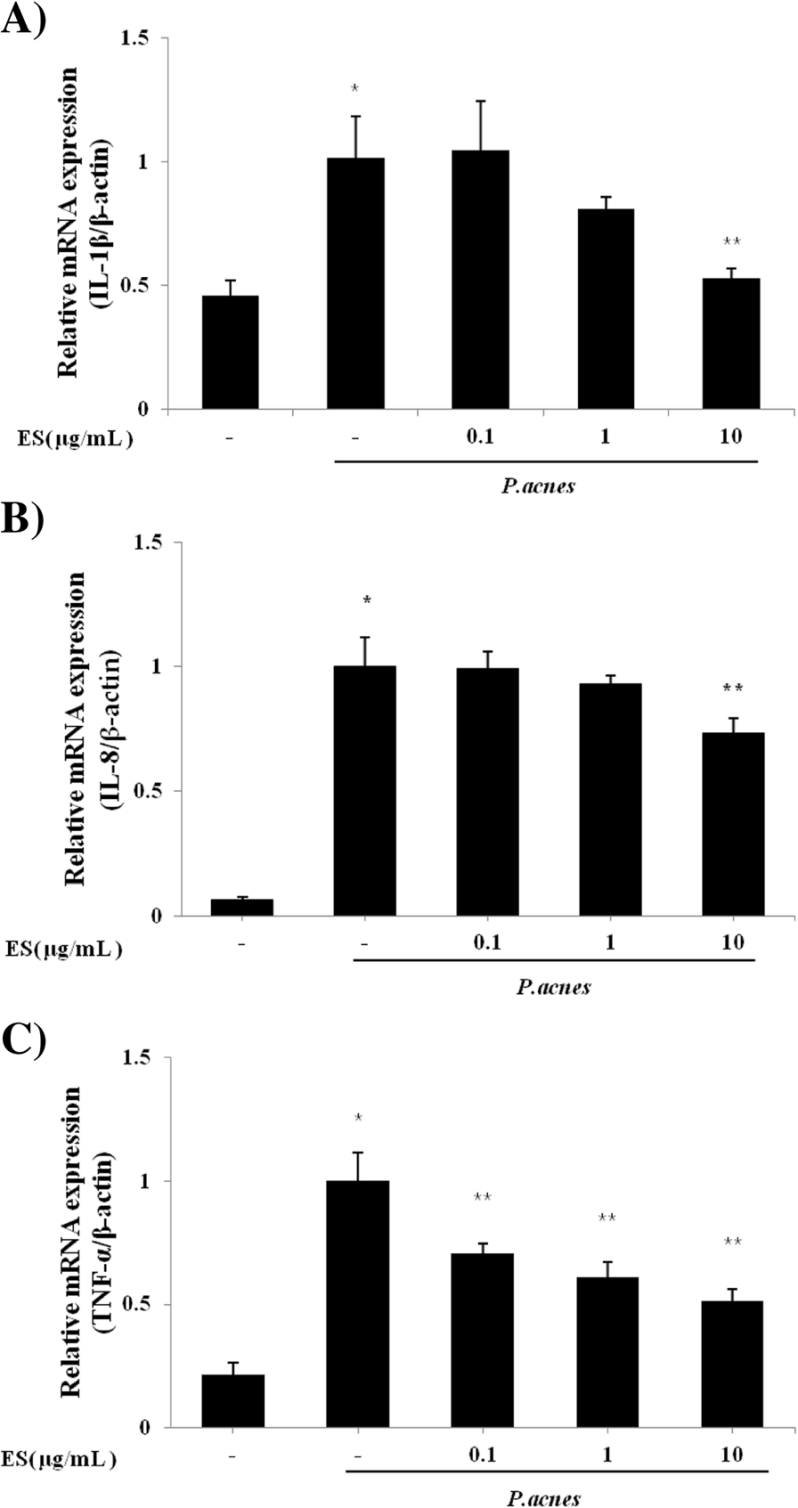
Effect of ES on the gene expression of (**a**) IL-1β, (**b**) IL-8 and (**c**) TNF-α in *P. acnes*-induced THP-1 cells. The expression level of mRNA was determined using a Real-time PCR. THP-1 cells were pre-treated with 0.1, 1 and 10 μg/ml of ES for 4 hrs incubation and then stimulated with *P. acnes* for 18 hrs incubation. Values represent mean ± S.E.M. Data were analyzed by Tukey post hoc test ( * *p* < 0.05 versus control and ** *p* < 0.05 versus P.acnes alone)

We also determined the mRNA expression levels of cytokines after ES treatment by real time RT PCR. Our results showed that ES suppressed the mRNA expression levels of TNF-α, IL-1β, and IL-8 in *P. acnes* induced THP-1 cells (Fig. 3).

### Regulatory effects of ES on activated MAPK signaling pathway in heat-killed *P.acnes*-treated THP-1 cells

**Fig. 4:**
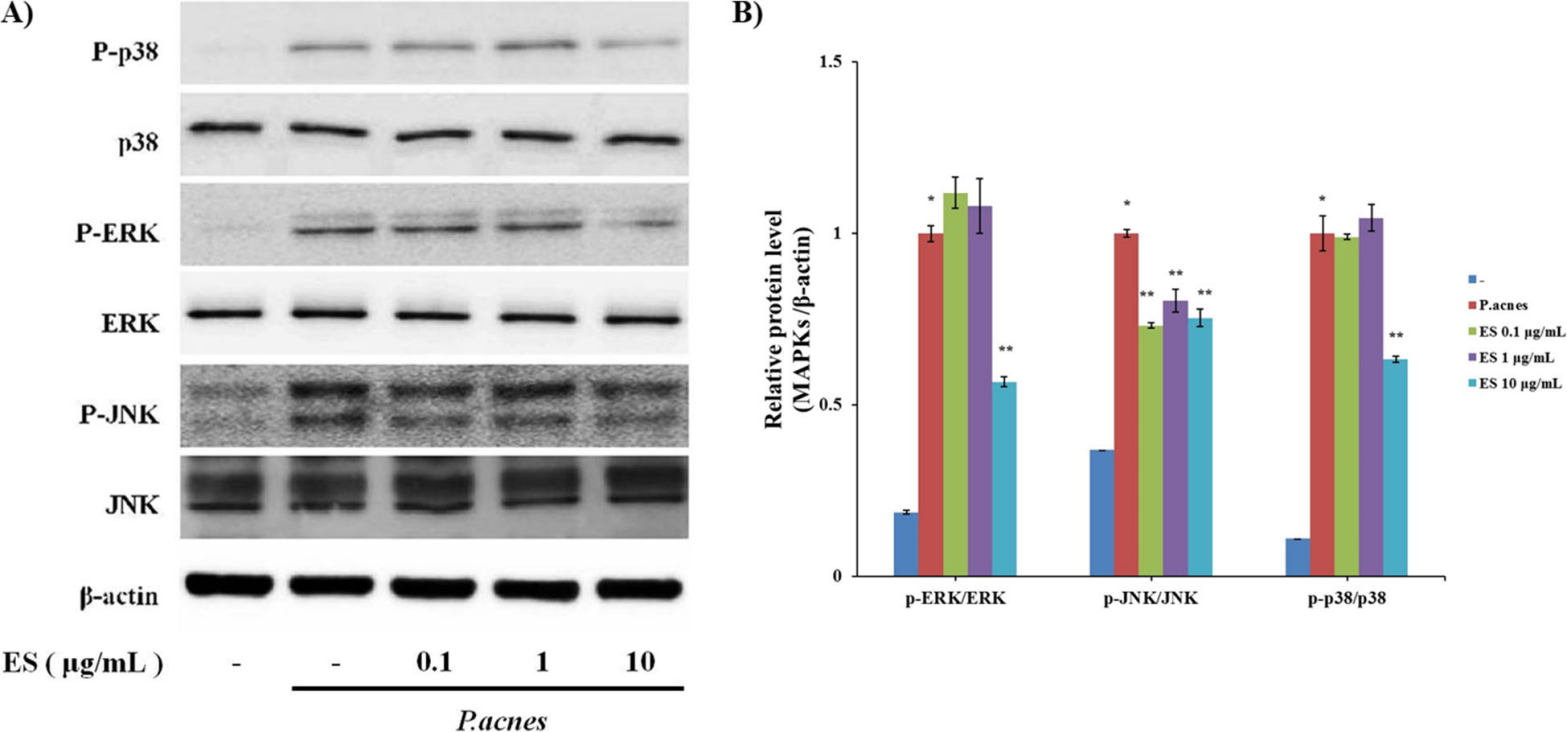
Effect of ES on the MAPK signaling pathway in *P. acnes*-indcued THP-1 cells. THP-1 cells were pre-treated with 0.1, 1 and 10 μg/ml of ES for 1 hrs incubation and then stimulated with *P. acnes* for 1 hrs incubation. (**a**) Western blot analysis shows that phosphorylation of p38, ERK and JNK is suppressed by ES treatment. (**b**) MAPKs/β-actin ratio were determined by densitometry. Values represent mean ± S.E.M. Data were analyzed by Tukey post hoc test ( *P < 0.05 versus non-treatment and **P < 0.05 versus *P.acnes* alone)

To determine the influence of anti-inflammatory properties of ES on MAPK signaling pathway, the levels of MAPK activation were examined by Western blotting analysis. As shown in Fig. 4, the phosphorylation levels of p38, JNK, and ERK were markedly increased in THP-1 cells treated with heat-killed *P.acnes*. However, ES treatment decreased *P. acnes*-induced phosphorylation of MAPKs such as p38, JNK, and ERK.

### Effects of ES on *P. acnes*-induced inflammation in vivo

**Fig. 5:**
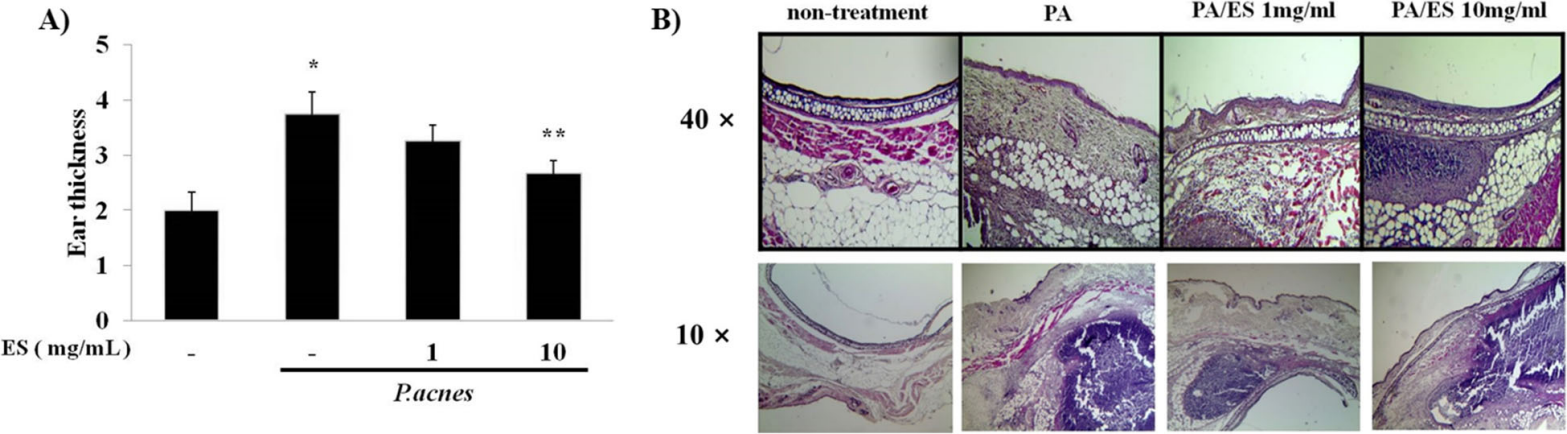
Effect of ES on ear thickness in living *P. acnes*-injected mice ears. (**a**) The suppress effects with 1, 10 mg/ml of ES on *P. acnes*-induced ear edema in mice were evaluated by measuring the ear thickness. (**b**) Paraffin sections of Ear tissue were stained with H&E observed by microscope. Values represent mean ± S.E.M. (*n* = 5). Data were analyzed by Tukey post hoc test ( * *p* < 0.05 versus control and ** *p* < 0.05 versus P.acnes alone)

To investigate the anti-inflammatory effects of ES on mice ears, live *P. acnes* were intradermally injected into mice ear. At 24 h post injection of live *P. acnes*, ES (1 mg/ml or 10 mg/ml) was injected into mice ears. At 24 h post ES injection, mice were sacrificed and ear thickness was measured by micro-caliper. The ear thickness of the *P. acnes*-treated group was increased 1.7 fold compared to that of non-treated group. Co-injection of 10 mg/ml of ES significantly reduced ear thickness (Fig. 5a). Inflammatory cells and thickness of epidermis were observed in H&E-stained section of *P. acnes*-injected ears. Intradermal injection with ES at 1 or 10 mg/ml significantly reduced the number of inflammatory cells and thickness of epidermis in a dose dependent manner (Fig. 5b).

### Chemical composition of ES

**Fig. 6:**
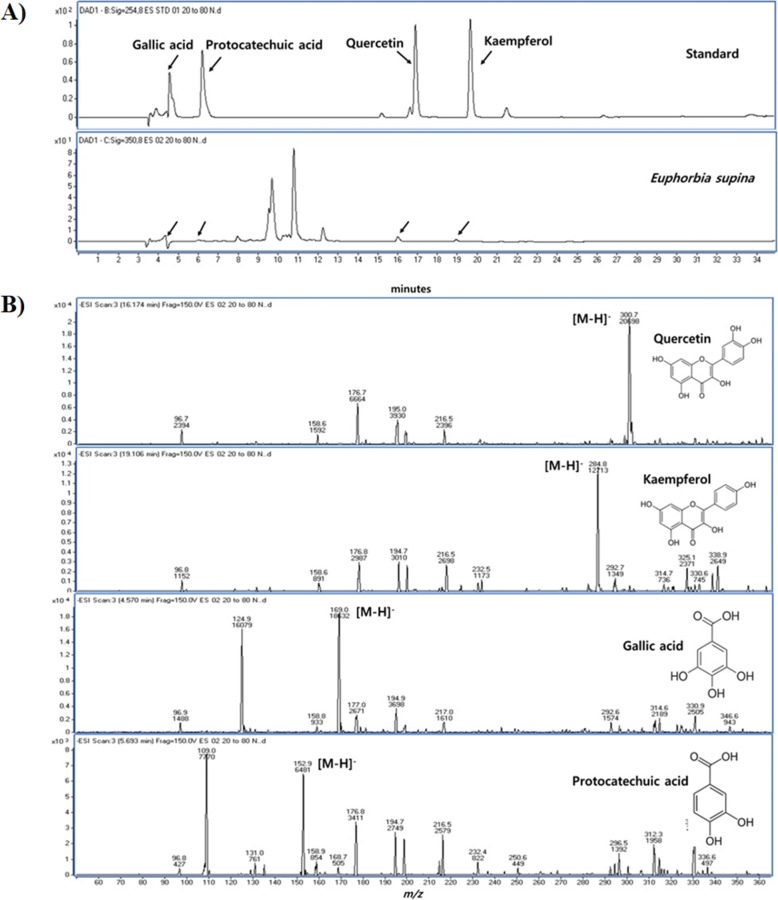
HPLC chromatogram of standards and *Euphorbia supina* (**a**), mass scan of quercetin, kaempferol, gallic acid and protocatechuic acid (**b**)

When analyzed with LC/MS system, the retention times of gallic acid, protocatechuic acid, quercetin and kaempferol were 4.5, 5.6, 17.0 and 19.8 min, respectively (Fig. 6). Quantitative analysis of quercetin and kaempferol gave a concentration of 4.480 mg/ml and 0.538 mg/ml in the extract. All of compounds mentioned above, were identified by retention time and molecular ion peak compared with standards.

## Discussion

*P. acnes* is one of the most abundant bacterium on the skin [2]. Although acnes is not an infectious disorder, the role of *P. acnes*, a Gram-positive bacterium that colonizes on the pilosebaceous unit, has been outlined in previous studies [11]. Injection of *P. acnes* into sterile keratinous cysts can lead to their rupture with consequent inflammation [12], thus providing evidence of inflammatory properties of *P. acnes.* In addition, heat-killed *P.acnes* can induce inflammatory response. Heat-killed *P.acnes* induced nitric oxide (NO) and IL-8 production in keratinocyte. Also, heat-killed *P.acnes* influenced activation of p38 MAP kinase [13]. In this study, *P.acnes* was used to induce inflammatory response including production of pro-inflammatory cytokines in THP-1 cells.

ES is a species of Euphorbiaceae traditionally used in eastern Asia for medicinal purposes [14]. ES is known to possess biologically active compounds. ES extract has compounds such as gallic acid, protocatechuic acid, nodakenin, quercetin, and kaemferol [8]. Especially, protocatechuic acid, gallic acid, quercetin, and laempferol were found as ingredients of ES (Fig. 6). Protocatechuic acid is known as having suppressive effect on TNBS-induced colitis [15], preventive effect on LPS-induced inflammatory response in fibroblast [16], and anti-oxidative effect [17]. In addition, gallic acid has several effects on allergic reaction [18], type 2 diabetes symptoms [19], oxidative stress, and hypertension [20]. These constituents (protocatetuic acid and gallic acid) might influence the regulatory effect of ES on *P.acnes*-induced ear inflammation.

Thus, this study investigated the potential of ES as an antibacterial agent for the treatment of acne vulgaris. However, ES has strong antibacterial activity. We observed that ES was nearly equally active against skin flora such as *Streptococcus aures* (JCM20624), *Propionibacterium granulosum* (KCTC5747), and *Staphylococcus epidermidis* (KCTC1917) (Additional file 1: Figure S1). Furthermore, the lipase activity of *P.acnes* is presented to have role of hydrolysis of sebum triglyceride to free fatty acids. In this process, acnes and skin inflammation is deepen [21]. For this reason, inhibition of lipase is can be a strategy to reduce skin inflammation. ES extract inhibited lipase activity of *P. acnes* (Table 1).

Several studies have reported that the anti-inflammatory effects of ES. ES extract has been reported to be able to reduce the levels of inflammatory mediators such as nitric oxide, IL-6, leukotrienes, and β-hexosaminidase [7]. Recent studies have declared that TNF-a and IL-8 can modulate inflammatory responses in monocytes [22, 23]. Our results showed that *P. acnes* induced secretion of TNF-a, IL-1β, and IL-8 in monocytic THP-1 cells. Moreover, ES treatments effectively inhibited the expression of these cytokines.

The fact that IL-1β is secreted in acne skin condition has proposed valuable effects of IL-1β-targeted therapy in patients suffering from anti-inflammatory acne-lesions [24, 26]. *P. acnes* is able to induce the secretion of chemokine CXCL8 in monocytes and keratinocyte [25, 26]. We also observed that the mRNA expression levels of cytokines in *P. acnes*-induced THP-1 cells were decreased by ES treatment. Our results showed that ES could reduce the expression levels of *P. acnes*-induced TNF-a, IL-8, and IL-1β at transcriptional level.

MAPK and NF-kB pathways have been proposed to be associated with *P. acnes*-induced inflammatory cytokine production [5]. MAPK signaling pathways can adjust cellular reaction to diffusion, differentiation, apoptosis, and inflammation in humans [27]. Previously studies have reported that melitin can suppress MAPK pathway in *P. acnes*-stimulated HaCaT keratinocytes [28]. We observed that *P. acnes* activated the phosphorylation of MAPK in THP-1 cells. Treatment with *ES* suppressed the phosphorylation levels of p38, JNK, and ERK induced by *P. acnes*.

Based on our in vivo results, we observed the anti-inflammatory effects of ES in *P. acnes*-treated animal model. Several studies have described that injection of live *P. acnes* can lead to the development of inflammatory skin disease in ear-inflammation model [26]. These studies have demonstrated that live *P. acnes* treated group has roughly 2 fold increase in ear thickness compared to PBS treated ear [26]. *P.acnes* can lead to accumulate immune cells such as neutrophil, monocyte, and eosinophil. Also, *P.acnes*-injected ear secrets IL-1β, MMP-2 and 9, and integrin α6 [29]. Our results also showed that injection of ES (10 mg/ml) significantly reduced ear thickness and the number of inflammatory cells.

In summary, our results demonstrated the antibacterial and anti-inflammatory effect of ES against *P. acnes* both in vitro and in vivo. ES significantly decreased the expression levels of various inflammatory cytokines in heat-killed *P. acnes*-treated THP-1 monocytic cells. In addition, *P. acnes*-induced inflammatory responses were inhibited by ES treatment through suppressing MAPK phosphorylation. Our results also showed that ES could inhibit *P. acnes*-induced inflammatory response in animal model. Our data suggested that ES extract could be used to treatment anti-inflammatory skin disease.

## Conclusions

ES extract has shown strong antibacterial activity against *P. acnes*. ES extract suppressed pro-inflammatory cytokines and MAPK signaling pathway. ES extract inhibited dermatitis in a mice model of acnes induced by intradermal injection of *P. acnes*. This study provides that ES extract might be used to treatment anti-inflammatory skin disease.

## Additional file

Additional file 1: **Figure S1.** The antibacterial effect of ES on skin microbe. To evaluate the antibacterial activity of ES extract against skin microbe, the strains were co-cultured with various concentrations of ES for 48 h. (A) *Staphylococcus aureus*, (B) *Staphylococcus epidermis*, (C) *Propionibacterium granulosum.* (DOCX 393 kb)
