# Supplementary material for: Inhibitory effects of Euphorbia supina on Propionibacterium acnes-induced skin inflammation in vitro and in vivo
Source: BMC Complement Altern Med. 2018 Sep 27;18:263. doi: 10.1186/s12906-018-2320-8 (PMC6161423; doi:10.1186/s12906-018-2320-8)
Supplement: Supplementary file 1 — Figure S1. The antibacterial effect of ES on skin microbe. To evaluate the antibacterial activity of ES extract against skin microbe, the strains were co-cultured with various concentrations of ES for 48 h. (A) Staphylococcus aureus, (B) Staphylococcus epidermis, (C) Propionibacterium granulosum. (DOCX 393 kb) [file 12906_2018_2320_MOESM1_ESM.docx]

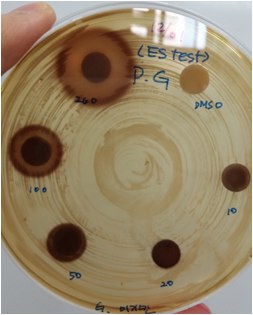

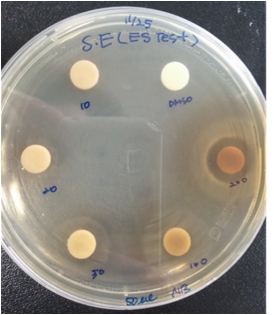

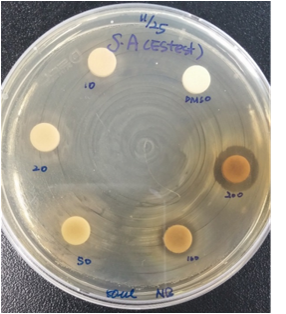
<Supplementary Data>

***S. epidermis***

***S. aureus***

***P. granulosum***

Supplementary figure. The antibacterial effect of ES on skin microbe.

To evaluate the antibacterial activity of ES extract against skin microbe growth, bacteria was co-cultured with various concentrations of ES for 48 hrs. (A) *Staphylococcus Aureus* (*S. aureus*), (B) *Staphylococcus Epidermis* (*S. epidermis*), (C) *Propionibacterium granulosum* (*P. granulosum*)
